# *Off-* and *Online* Heterosexual Dating Violence, Perceived Attachment to Parents and Peers and Suicide Risk in Young Women

**DOI:** 10.3390/ijerph17093174

**Published:** 2020-05-02

**Authors:** Marcela Gracia-Leiva, Alicia Puente-Martínez, Silvia Ubillos-Landa, José Luis González-Castro, Darío Páez-Rovira

**Affiliations:** 1 Department of Social Psychology and Methodology of Behavioral Science, Faculty of Psychology, University of the Basque Country, 20018 Donostia-San Sebastián, Spain; mgracia006@ikasle.ehu.eus (M.G.-L.); alicia.puente@ehu.es (A.P.-M.); dario.paez@ehu.es (D.P.-R.); 2 Department of Social Psychology, Faculty of Health Science, University of Burgos, 09001 Burgos, Spain; 3 Department of Social Psychology, Faculty of Educational Science, University of Burgos, 09001 Burgos, Spain; jlgoca@ubu.es

**Keywords:** dating violence, cyber dating violence, women, adolescence, suicide risk, peer attachment, parent attachment

## Abstract

Dating violence (DV) is a public health problem among young people, especially women. It involves violent acts towards one’s partner and occurs face-to-face (offline) or through the Internet (online). Offline DV is linked to suicidal ideation and attachment to parents and peers. Fewer studies analyze the psychological and social consequences of online DV. This study tests the link between young women’s DV victimization (off- and online), suicide risk (SR), and parent and peer support in a sample of young Spanish females (N = 1227) (*M*age=19, *SD* = 2.82; range = 13–28). Results confirm that compared to non-victims off- and online DV increase suicidal thoughts and attempts. This effect is stronger for victims of both types of DV (thoughts: OR offline DV = 3.11; CI95% 2.06, 4.69; OR online DV = 2.37; CI95% 1.69, 3.32; OR off-online DV = 4.19 CI95% 2.44, 7.17) (attempts: OR offline DV = 4.02; CI95% 1.83, 8.81; OR online DV = 3.69; CI95% 1.96, 7.01; OR off-online DV = 10.55 CI95% 2.56, 44.43). Mediation and moderation models were used to assess the effect of perceived attachment of parents and friends in DV victims and SR. Mediation analyses indicated that perceived attachment and proximity to parents and peers reduces the impact of DV on SR. Moderation analyses showed that a high level of perceived peer attachment reduces the effect of offline DV on SR. Regarding off-online DV, a high level of perceived parent attachment mitigates suicide risk. Loneliness, lack of care from loved ones, and thwarted belongingness increase suicidal thoughts in DV victims. Peers and parents’ proximity may prevent risk behaviors in DV victims.

## 1. Introduction

Dating violence (DV) is a social and public health hazard and refers to any physical, sexual or psychological aggression inflicted by a member of a couple against the other. Adolescent or young adults’ DV has been defined as a type of violence that occurs in romantic relationships with different degrees of formality between early adolescence (10 years) and early adulthood (up to 30 years of age) [1,2,3]. Some authors indicate that DV occurs in couples who do not live together and have no children in common or legal ties [4,5]. Abuse of one’s dating partner may occur face-to-face (offline), but it can also happen on the Internet, using new technologies and social networks (online). Offline DV comprises intentional abuse or sexual, physical or psychological acts from one partner to another [1]. Online DV includes psychological control, harassment and direct aggressive behaviors and has a negative impact on victims [6,7,8,9,10]. 

Recent studies have found that off- and online DV are related [11,12]. Online DV overlaps with psychological abuse [6], physical and sexual DV [13] and stalking [14]. Regarding offline DV, studies have also found positive correlations between different forms of victimization (verbal, physical and relational) [15]. However, online DV also differs from offline DV. In online DV, harassment may have a higher scope and visibility, but also a higher risk of non-disclosure of the abuse due to its private nature [16] as well as a greater probability of repeated victimization since social networks are permanently updated [17]. Moreover, online DV exposes the victim mediatically during the relationship, or even after it is over [18].

The empirical evidence on sex differences in the simultaneous prevalence and frequency of off- and online DV is scarce. Regarding offline DV, a recent systematic review revealed that victimization mainly affects females compared to males [1]; however, other authors have not found a difference in victimization between sexes [19]. Rates of offline sexual and physical women’s DV victimization varied between 17% (a national survey in the United States) [20] and 88% [21]. Indeed Smith et al. [21] found that during adolescence young women were at greater risk of suffering physical and sexual assault from their partners than young men. 

As for cyber dating violence, there is also a large variability in prevalence rates. A study reported rates of 76.5% (females) and 77.1% (males) in the United States, indicating that males stated more electronic victimization and females reported more anticipated distress when suffering cyber DV [22]. However, Stonard [23] found that cyber DV was prevalent both among females (victimization: 12–57% at least once or more in the past year) and males (victimization 11–54%). Nevertheless, females had a greater likelihood of being identified as victims in online sexual DV. Similarly, international reports have found higher rates of cyber DV victimization in girls than boys [24]. A multi-country study conducted in Europe concluded that young women (between the ages of 18 to 29 years) are at a heightened risk of being exposed to different types of cyber violence [25] and that one in ten women had suffered some cyber violence since the age of 15 [26]. In Spain, studies found rates of online victimization ranging from 3.5% (e.g., had shared images of themselves without their consent) to 9.2% (e.g., I’ve received messages on the Internet insulting me) [27]. Specifically, a study showed higher rates of DV control behaviors towards women (80.4%) than direct aggression (29.6%) through social networks [7]. 

### 1.1. Suicidal Risk (SR) and Adolescent/Young Adults Dating Violence

Suicide attempts and suicidal ideation are a public health priority. According to the World Health Organization [28,29], suicide is the second leading cause of death among people between 15 and 29 years of age. A study reported that both suicidal ideation and suicide attempts in adolescent samples are greater in girls than in boys and increase with age [30]. In Spain, two representative studies analyzed rates of suicide risk. The first study indicated that women had a higher prevalence of suicidal ideation, but no previous attempts, compared to men [31]. The second study found that women are more likely than men to have previously attempted suicide [32]. In addition, a revision of longitudinal studies (adolescents and young adults) found that being a victim of DV is one of the specific risk factors for taking one’s life in women [33].

The Interpersonal Theory of Suicide [34,35] has been cited to explain suicide risk in DV samples [36,37]. This theory postulates that frustrated interpersonal needs (frustrated belonging and perceived burden) are antecedents to suicidal ideation. According to this theory, suicidal ideation is the result of feelings of responsibility and self-hatred (perceived burden) and feelings of loneliness and low mutual attention (frustrated belonging) [38]. Both psychological and physical aggression have the potential to promote frustrated belonging and perceived burden. Furthermore, this theory proposes that the acquired capacity to act on the desire to take one’s life develops through previous exposure to painful and fear-inducing experiences. Accordingly, experiences of physical and sexual violence could be painful or fear-inducing physical experiences. Moreover, this is an immediate antecedent to suicidal ideation [38].

Therefore, suffering DV (offline) has been associated with suicide and suicidal ideation in women [39]. A multi-country study (21 countries) with university students concluded that there was no correlation between males’ suicidal ideation and DV victimization (except for physical violence), while suffering any type of violence was associated with higher rates of suicidal thoughts in women [40]. Olshen et al. [41] found that DV (during the past 12 months) was associated with suicide attempts in adolescent girls. Furthermore, two meta-analytical studies supported these results. One study included adolescent and young adults, men and women, showing an increased risk of suicide attempts for victims of DV compared to their non-exposed counterparts [42]. In a second meta-analysis of longitudinal studies, two studies with adolescent and young women found DV was associated with attempted suicide [43].

Studies have also documented the consequences of different types of cybervictimization on women, confirming that it is associated with increased negative feelings, social avoidance and suicide attempts [44]. Online DV has been negatively related with well-being and is a significant negative predictor of self-esteem and a positive predictor of emotional distress [45]. In Spain, Borrajo and Gamex Guadix [7] found that online DV victimization was associated with increased symptoms of depression and anxiety in adolescents. However, fewer studies have been conducted regarding online DV in women and risk of suicide.

### 1.2. Social Context, Perceived Attachment to Parents and Peers and Adolescent/Young Adults Dating Violence

According to attachment theory, peers and parents are the most important figures during adolescence and provide emotional support when needed [46]. Attachment figures are those that teens feel they can count on in times of increased stress or danger [47]. Two meta-analyses confirm that high attachment to peers is positively correlated with lower indices of offline DV victimization [48,49]. Moreover, adolescents who reported a high level of attachment to their parents also reportedly suffered less offline DV [49].

Supporting these results, victims of offline DV (both genders) showed lower levels of social support from friends and family compared to those who were not victims. However, social support given by peers was only related to lower levels of DV victimization among girls but not for boys, while parental social support was not been associated with DV victimization. These results suggest that adolescents rarely turn to their parents or other adults for concerns and issues related to DV and indicate that friends may play a crucial function as protective figures in DV, mostly among girls [50,51]. Nevertheless, another study found that adolescents who suffered online DV tended to seek support first from parents than from peers or teachers, especially in the case of girls [52]. 

### 1.3. Social Context, Perceived Attachment to Parents and Peers and SR

Regarding emotional problems, support from parents and friends has been defined as two relatively independent support systems. During early adolescence, the search for parental support decreases and peer support increases because it is during this period that adolescents start to establish intimate relationships outside of the family and want to become more independent from their parents’ guidance. However, this autonomy is frequently still established within the context of continuing close and trusting relationships with parents, and the lack of parental support remains the best sign of mental problems during adolescence [53]. In this sense, Mackin et al. [54] found that high levels of parental support protected adolescent girls from developing suicidal symptoms following a stressor event. This effect was less pronounced for peer support. The global importance of attachment with parents has also been mentioned by authors such as Sternberg et al. [55] stressing that this attachment is positively correlated with measures not only of family cohesion and expressiveness, but also with higher self-esteem, life satisfaction, and lower levels of psychological symptomatology, such as distress, depression, anxiety, resentment, covert anger, or loneliness.

As suggested above, both attachment with parents and peers have been addressed as predictors of suicide and risk factors for DV. Previous findings indicated that parent–child relationships marked by emotional distance, non-responsiveness, and greater conflict are associated with more risk-taking behaviors and DV in adolescents [56]. Conversely, girls with secure perceived peer support may have some type of protection from engaging in violent relationships [51]. Also, a study found that different aspects of mothers’ parenting control protect against various forms of victimization in DV [57]. 

### 1.4. The Current Study

Prior studies have examined many of these correlates individually, but there is scarce research addressing these factors simultaneously. Although the literature confirms the bi-directional pattern of DV [58,59], results systematically show differences between men and women in severity and consequences [2]. For example, a meta-analysis [33] that confirms the relationship of DV victimization with suicide attempts, is significant only in women. Consequently, this study is focused on studying DV and suicide risk from the perspective of female victims. Furthermore, several authors have highlighted the importance of studying violence in intimate relationships and DV from a gender-specific approach, considering gender-specific risk factors and consequences associated with violence [60,61]

The first aim of this study was to examine the prevalence of off-online DV and suicide risk. Second, this study analyzes the relationship of off-online DV, perceived attachment to parents and peers and suicide risk. We expected to find that offline and online DV were positively associated with SR and negatively with perceived attachment to parents and peers. Therefore, those with poor attachment styles would be more likely to experience riskier behaviors (DV and SR). Third, this study will examine whether parent and peer support mediate and/or moderate the relationship between only offline or online DV, and simultaneous off-online DV on SR. We expected to find that stronger attachment to parents and peers would have a buffering effect between offline and online DV and SR. 

## 2. Materials and Methods 

### 2.1. Sample

We conducted a quantitative study using a cross-sectional design. Data was obtained by convenience sampling. The sample was composed of *N* = 1227 females, aged 13 to 28 (*M* = 18.76, *DT* = 2.82), who have, or have had, a dating relationship with a male partner, are not living together and have neither children or any binding legal ties. Of the total sample group 91.5% were Spanish, 5.5% from Latin-America, 1.7% from Europe, and 0.7% indicated “others”. Moreover, 0.6% did not answer this question. A total of 88.7% (*n* = 1088) of the participants had been involved in a relationship in the past and 58.7% (*n* = 720) were involved in one at the time of the survey. Their first intimate relationship had begun approximately when they were 15 years old (*M* = 15.33, *DT* = 2.41). 

### 2.2. Procedure

Questionnaires were administered online (21.8%) and through pen and paper (78.2%) in 10 secondary schools and 12 universities in Spain. Three researchers (two psychologists and a social educator) visited the centers to collect the information. The questionnaire was answered, with collaboration and assistance from the teachers, during tutoring in classes and took approximately 30-40 minutes to complete. Moreover, the questionnaire was disseminated through the Qualtrics platform, and a link was sent via email. The study has received full approval by the ethics committee of the University of Burgos (IR 20/2019) meeting the ethical research criteria with human beings of the Helsinki declaration, and assuring anonymity, confidentiality, respect of privacy and voluntary participation. The final sample only included participants who were currently in a dating relationship or those who had been in a dating relationship, and in both cases, a minimum of a one-month relationship was required.

### 2.3. Measures/Instruments

(a) Cuvinova [62]. This instrument measures frequency of suffering or having suffered offline violence from a partner in intimate relationships. It includes 20 items and five dimensions with four items in each dimension: Detachment (e.g., *Stops talking to you or disappears for several days, without giving any explanation, as a way of showing his anger*) (α = 0.788); Humiliation (e.g., *criticizes you, underestimates you or humiliates your self-esteem*) (α = 0.824); Coercion (e.g., *talks to you about relationships he imagines you have*) (α = 0.816); Physics (e.g. *He hurts you with some object*) (α = 0.956) and Sexual violence (e.g., *Insists on touching that isn’t pleasant for you and that you don’t want*) (α = 0.970). The response range of the scale was between 0=never to 4=almost always. The total score of the scale is calculated by adding the mean of each of the five dimensions. Higher scores indicate more offline DV victimization.

(b) Cyberdating Abuse Questionnaire (CDAQ) [6]. It consists of 20 items that collect information about frequency of victimization and perpetration of various types of cyber DV (ICT and social networks). It comprises two dimensions: direct aggression (e.g., *Sending and/or uploading photos, images and/or videos with intimate or sexual content without permission*) and control and monitoring (e.g., *Checking social networks, WhatsApp or email without permission*). In this study, only the victimization scale was used. The questionnaire is answered on a 6-point Likert scale that asks how many times the behaviors have occurred during the last year: 1 (never), 2 (not in the last year, but it happened before), 3 (rarely: 1 or 2 times), 4 (sometimes: between 3 and 10 times), 5 (often: between 10 and 20 times) and 6 (always: more than 20 times). The internal consistency for direct aggression was α = 0.826, and for control, α = 0.940. Additionally, in order to create the prevalence scores, CDAQ was recoded into 0: no abuse behavior (non-victims) and 1: one or more abusive behaviors (victims). The total score of the scale was obtained by adding the mean of each of the two dimensions. Higher scores indicate a higher frequency of online DV victimization.

(c) A reduced version of the Inventory of Parent and Peer Attachment Scale (IPPA) was used [63,64]. It contains 24 items that assess the level of security felt by the adolescent toward significant attachment figures (peers and parents). Both the parent (IPPA-P) (α = 0.87) and friends/peers (IPPA-F) (α = 0.81) attachment subscales contain 12 items. Both IPPA subscales include three dimensions: a) trust or confidence (e.g., *When I´m angry about something my parents try to be understanding/ My friends listen to what I have to say*); b) communication (e.g., *I tell my parents about my problems and troubles/ My friends are concerned about my well-being),* and c) alienation (e.g., *Talking over my problems with my parents makes me feel ashamed or foolish/ I feel alone or apart when I am with my friends)*. Dimensions of trust and communication suggest an accepting environment provided by parents and peers. The scale ranged from 0 = almost never or never, to 4 = always. The total scale score was calculated by adding the results from the communication and confidence scale and subtracting the score from the alienation scale. Higher scores indicated a greater perceived attachment to parents and friends. As a whole, these dimensions account for an adolescent’s ability to ask for and seek help from parents and/or friends in difficult circumstances.

(d) The Spanish Suicide Risk Scale (SRS) [65,66]. The scale consists of 15 items with a dichotomous response (Yes = 1, No = 0). It includes questions about symptoms of depression and hopelessness, previous autolytic attempts, suicidal ideation and other aspects related to the risk of suicide attempts. An exploratory factor analysis (EFA) showed four components that explained 49.60% of the cumulative variance. The analysis confirms that the first factor included items 13, 14, and 15 and explained 14.29% of the variance. Factor 2 included items 4, 5, 7 and 9 (13.73% of the variance); factor 3: items 3, 6, 8, 10 and 12 (12.94% explained variance) and factor 4: items 1 and 2 (8.64% of the variance). The CFA also confirmed the four factor model indicating a good fit for the data: CFA = 0.94, TLI = 0.93, RMSA = 0.046, IC95% [0.040, 0.052]. Item 11 was deleted due to it not reaching a.30 factor loading threshold. On the basis of this result and the specific concept under study, only 3 items of the scale related to suicidal ideation and suicide attempts were used: 13 (*Have you ever thought about suicide*?), 14 (*Have you ever told anyone that you would take your own life*?), and 15 (*Have you ever tried to take your own life*?). The prevalence analyses were then performed considering affirmative responses for items 13, 14 and 15 separately. We also calculated the total score of the suicide risk variable considering the sum of these three items.

### 2.4. Data Analysis

There are no significant differences between the pen and paper and online questionnaire application in SR (*t*_(1225)_ = 0.283, *p* = 0.777), offline (*t*_(1224)_ = 1.299, *p* = 0.194) and online (*t*_(1225)_ = 0.779, *p* = 0.436) violence, and peer (*t*_(1225)_ = 0.938, *p* = 0.349) and parent (*t*_(1225)_ = 0.048, *p* = 0.962) attachment. Thus, data analyses were carried out jointly. To obtain the percentage estimation of online, offline and joint off-online DV, the sample was split into victims and non victims regarding at least one episode of DV. Descriptive statistical analyses were applied to describe the sample and prevalence of DV and SR. Furthermore, to analyze the relationship between DV, SR and perceived attachment to parents and peers, partial correlations (*r_p_*) were conducted including age as a control variable.

To test the hypothesis of the moderating and mediating effects of IPPA-P and IPPA-F on DV and SR, the PROCESS macro for SPSS v.25.0 (IBM Corp 2007, Armonk, USA) was used [67]. To examine the mediation effects, three models were estimated (PROCESS model 4). One for each independent variable due to their high collinearity (offline and online) and one for the joint effects of both variables (off-online). The indirect effect, standard errors (SE) and confidence intervals (CI, 95%) based on the distribution obtained with the bootstrap method set to 10.000 iterations were estimated [68]. To examine moderation effects three models were also estimared (PROCESS model 1). The moderation analysis will allow us to understand the attachment levels (high, medium and low) in which dating violence increases or decreases suicide risk. The conditional effect, standard errors (SE) and confidence intervals (CI, 95%) were estimated with the bootstrapping samples method set to 10.000 iterations. A conditional indirect effect is considered significant if the confidence interval (CI at 95%) does not include the value 0. The PROCESS macro interprets significant interactions at the 16th, 50th and 84th percentiles, of perceived attachment to parents and peers as potential moderating effects [67]. In both analyses, off, online and off-online DV will be entered as a categorical independent variable (0 “not victim” and 1 “victim”), perceived attachment to parents and peers as a continuous moderator or mediator, and suicide risk as the dependent variable. Age was included as a control variable. 

## 3. Results

### 3.1. Prevalence Rates of Off and Online Dating Violence and Suicide Risk

Overall, 76% of teenage girls indicated that they had experienced some form of offline DV violence, and 68.8% reported online DV (*χ^2^* = 221.97, *p* = 0.0001). Moreover, 56.8% experienced both online and offline DV. Almost two-thirds of the participants reported being a victim of DV by detachment or monitoring/control. Just over half of the participants reported experiencing coercive violence and approximately one-third of them indicated having experienced at least one direct aggression through the Internet, as well as humiliating behaviours and sexual assaults. Around 11% reported experiencing physical abuse. Regarding suicide rates, 22.7% of the participants informed about suicidal ideation, 11.2% talked to someone about suicide, and 8% attempted to take their own life (See Table 1).

We also conducted a chi-square test to analyze whether victims of offline and online violence thought about and attempted suicide more than non-victims. In both cases, online and offline DV, frequencies of suicidal ideation and suicide attemtps were significantly higher among victims compared to those who did not suffer violence (see Table 2). These patterns were repeated among all dimensions of the CUVINOVA scale and the Cyberdating Abuse Questionnaire. Victims of offline DV show a three to four times higher risk of suicidal ideation and risk of attempted suicide compared to non-victimized women. Women who suffer sexual violence have the highest risk of thinking about suicide and those who suffer physical violence show the highest risk of suicide attempts. Online DV victims versus non-victims show 2.37−3.69 times higher risk of suicidal ideation and risk of suicide attempts, with direct aggression the factor that increases the most both thoughts and attempted suicide. Regarding joint off-online DV victims, versus non-victims, results show a 4.19 times higher risk of suicidal thoughts, and a 10.55 times higher risk of attempted suicide (see Table 2). 

### 3.2. Relationships between Off and Online DV, Perceived Attachment to Parents and Peers, and SR

Partial correlation analyses, controlling age (Table 3), shows that offline and online, as well as joint off-online DV were positively associated. Moreover, there was a significant and positive relationship between all types of DV (off- and online) and suicide risk. Also adolescent off and online victimization (total scale scores and dimensions) was negatively and significantly related to comunication and trust in the IPPA-P and the IPPA-F scores and positively associated with parental and peer alienation. Physical violence was not significantly related with the IPPA-P communication dimension. Monitoring/control was neither related to the general IPPA-F score or trust. The IPPA-F’s communication dimension is negatively related only to general offline DV, humilliation and sexual violence. Parental and peer perceived attachment are positively related among them. Similarly, the risk of suicide was negatively related to communication and trust and positively with alienation from parents and peers.

### 3.3. Perceived Attachment to Parents and Peers as A Protective Factor against SR in Female Adolescent Victims of Off- and Online DV

**Figure 1:**
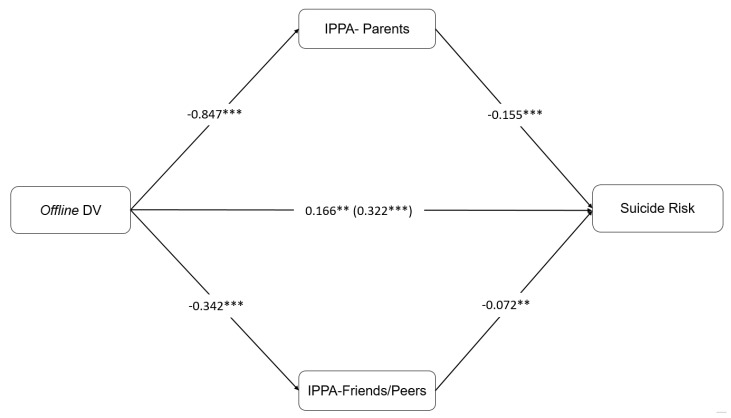
Model 1 with IPPA–P and IPPA–F as a Mediation in the Effect of Offline DV on Suicide Risk.

Three mediation analyses were carried out to check whether perceived attachment to parents and peers mediated the relationship between having suffered off, online, and joint off-online DV and the risk of taking their own life. Age was included as a covariate in the analysis. As shown in Figure 1, offline DV had a direct and positive effect on SR (F = 55.093 *p* = 0.0001) and IPPA-P (c1) and IPPA-F (c2) had a significant negative effect on SR. The indirect effects indicated that IPPA-P (b = 0.1319, SE = 0.0215, 95% CI [0.0938, 0.1779]) and IPPA-F (b = 0.0248, SE = 0.0094, 95% CI 0.0098, 0.0474) explained the relationship between DV and SR. The model explained 16% of the total variance. Therefore, suicide risk was reduced when young and adolescent women found more confidence, communication and less alienation from their parents and peers. 

**Figure 2:**
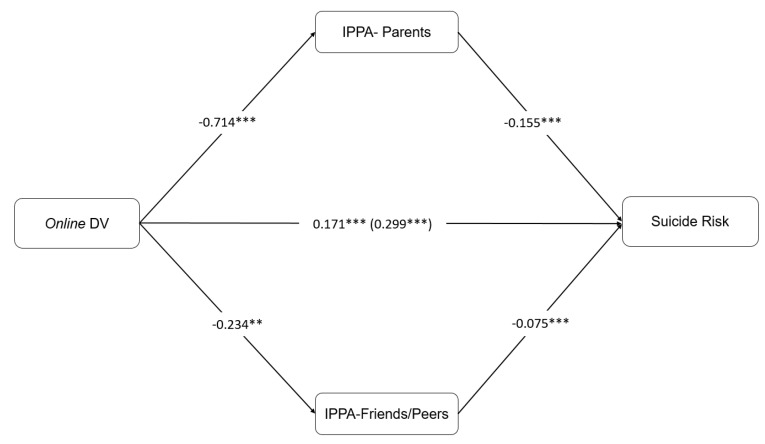
Model 2 with IPPA–P and IPPA–F as a Mediation in the Effect of Online DV on Suicide Risk.

The contrast of indirect effects analyses were also significant (c1–c2: b = 0.1567, SE = 0.0235, 95% CI 0.1147, 0.2064). This implies that victims who have more family support (high quality attachment relationships) reduced the effects that offline violence had on SR in comparison to peer support. Model 2 with online DV was also significant (F = 56.283, *p* = 0.0001). Results indicated that online DV had a significant and positive direct effect on SR. Moreover, perceived attachment to parents and peers was also associated with lower SR. Indirect effects for IPPA–P (b = 0.1110, SE = 0.0192, 95% CI [0.0772, 0.1530]) and IPPA–F (b = 0.0176, SE = 0.0078, 95% CI [0.0055, 0.0369]) were significant. The model explained 16% of the variance. The comparison between indirect effects was also significant (c1–c2: b = 0.1285, SE = 0.0216, 95% CI [0.0889, 0.1730]), indicating that perceived attachment to parents had a higher effect than attachment to peers in reducing the effect of online DV on SR (see Figure 2). 

**Figure 3:**
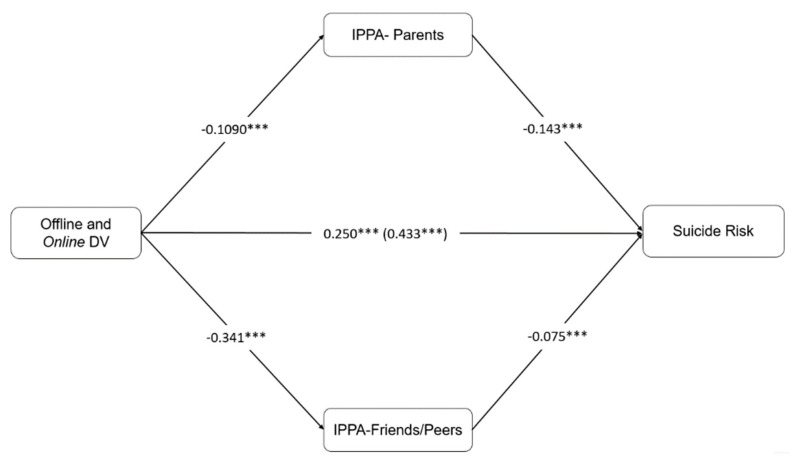
Model 3 with IPPA–P and IPPA–F as a Mediation in the Effect of off-online DV on Suicide Risk.

Model 3 including being a victim of both offline and online DV was also significant (F = 42.136, *p* = 0.0001). Results indicated that joint off-online DV had a significant and positive direct effect on SR. Perceived attachment to parents and peers was again associated with lower SR. Indirect effects for IPPA–P (b = 0.1567, SE = 0.0270, 95% CI [0.1096, 0.2152) and IPPA–F (b = 0.0257, SE = 0.0111, 95% CI [0.0083, 0.0528]) were significant. The model explained 16% of the variance. The comparison between indirect effects was also significant (c1–c2: b = 0.1310, SE = 0.0290, 95% CI [0.0798, 0.1923]), indicating that perceived attachment to parents once more had a higher effect than attachment to peers in reducing the effect of joint off-online DV on SR (see Figure 3).

Moderation analyses were applied to examine at what levels of IPPA–P and IPPA–F, the effect of DV in female adolescents and young adults did not increase suicide risk. Three models were estimated, one for each independent variable (offline, online, and off-online) to reduce collinearity. As shown in Table 4, significant direct effects emerged for offline DV and IPPA–P, but not for IPPA–F. The moderation analysis showed a significant interaction effect between offline DV and IPPA–F. By examining the conditional indirect effects of offline DV (0 = Non victim, 1 = Victim) on suicide risk at the three levels of IPPA–F (Low, Medium and High), results revealed that at a high level of perceived attachment to friends, the effect of offline DV on suicide risk was non significant. Also, at a medium level of IPPA–F when the perceived attachment to parents was high, the effect of offline DV on suicide risk is non significant. Therefore, only when victims of offline DV have high perceived attachment to friends, or medium but parents’ support is high, does suicide risk not increase. However, at low and medium levels of IPPA–F, the effects of offline DV on suicide risk were significant. Thus, suicide risk increases in female offline victims when the perception of support from friends is low or medium. 

In contrast, the interaction effects between online DV, IPPA–P and IPPA–F were non significant. Therefore, perceived attachment to parents and friends does not have a moderating effect. Main effects indicate that female online DV victims are at greater risk of suicide than those who are not victims. Also, as the perception of attachment to parents and friends increases, the risk of suicide decreases (Table 5). 

Table 6 shows significant direct effects for off-online and IPPA–P. In this model, the moderation analysis revealed a significant off-online DV x IPPA–P interaction effect on suicide risk. By examining the conditional indirect effects for off-online DV (0 = No victim, 1 = Victim) on suicide risk at the three levels of IPPA–P (Low, Medium, High), results reflected that at a high level, when the perceived support of friends is high or medium, the effect of off-online DV on suicide was non significant. So, in victims of off-online DV who have high perceived attachment to parents and medium or high perceived attachment to friends, suicide risk does not increase. At a low and medium level of IPPA–P, the effect of off-online DV on suicide risk was significant and positive. In these cases, suicide risk increases in female DV victims.

## 4. Discussion

This study analyzed the prevalence of off and online DV and suicide risk in a sample of Spanish teenage/young women. We aimed to explore the relationship between off and online DV, perceived attachment to parents and peers and suicide risk. In addition, we explored the link between attachment, and its buffering role, in the relationship between off and online DV and SR. 

The findings of this study show that there was a high prevalence of offline and online DV in adolescent and young adult girls. The percentage of face–to–face violence was higher than in online DV. These results seemingly contradict a British study with adolescents (boys and girls) in which cyber DV was more prevalent than offline DV (controlling and physical violence) [23]. In contrast, in this current study, rates of offline DV violence reached 76%. Rodríguez–Franco et al. [69] found similar results in a sample of Spanish adolescents, showing rates of 70%. López–Cepero, Lana, Rodríguez Franco and Rodriguez Díaz [70] reported lower rates of offline DV in young Spanish girls (between 2.3% and 27%) (15 and 25 years) than the percentages found in this study (between 11% and 65% depending on the dimension). We also found that psychological violence (detachment, humiliation and coercion) was the most common type of DV with a prevalence of between 65% and 36%, in line with results from López–Cepero et al. [70].

Online DV was present in 69% of cases. The most frequent types of violence were monitoring and control violence, and to a lesser extent, direct aggression. These findings are in line with those mentioned by Borrajo and Guadix [7] in a study carried out with a Spanish adolescent sample that used the same measures and with studies conducted in various countries [12]. Furthermore, prior longitudinal research also indicated that offline (psychological and physical) and online victimization DV were positively related [71]. More than half of the adolescent girls in this study reported experiencing both off-online DV (57%). This result coincides with previous findings and suggests that DV does not tend to occur in isolation and that different types of violence are interrelated and coexist in courtship [12,13]. Moreover, a recent study found that different forms of offline DV victimization were a predictor of online DV [72]. These results also suggest that technology and social media may provide new opportunities for online DV victimization, which may not have been possible before the development of the internet and social media. Moreover, results confirm that new technologies can be used to connect with a romantic partner but also to control and humiliate them privately and publicly [13]. Thus, DV experienced by young women in digital spaces can continue in real life and vice versa.

Regarding suicide ideation rates, 22.7% of girls reported thinking of suicide after DV. These results are consistent with the percentage of SR (23.1%) found in a male and female Spanish sample with similar characteristics [73]. Moreover, results indicated that around 11.2% of DV victims talked to someone about the idea of taking one’s own life, and 8% had attempted suicide after suffering DV. These results show higher rates of suicide ideation and attempted suicide (9.7% and 5.6% respectively) than the previously mentioned study [73]. 

Specifically, our findings confirm that the percentage of young women who thought about suicide or attempted suicide is higher among those who suffered offline and online DV compared to non–victims. This is especially the case in those young women who have suffered both types of DV. Suicidal ideation was approximately between two and three times higher for those who reported suffering offline and online DV, and over four times higher in the joint DV situation. In addition, the likelihood of attempting suicide was 3.5 times higher for those girls who suffered online DV and four times higher in those who suffered offline DV compared to non–victims. Nevertheless, an even stronger burden lays once again on those women who experience both types of DV. In this case, there is a tenfold increase in the risk of taking one’s own life. All these results were supported by data from the correlational analyses. Correlations confirm that DV (off, online and off–online) are closely linked to an increase in SR rates among adolescent girls. These results are consistent with other studies conducted with women confirming that victims of DV show more suicidal ideation [39] and attempted suicide [33,43].

These results also lend support to the interpersonal theory of suicide [34,35]. Chu et al.’s [74] meta–analysis posits that the interaction between frustrated belonging and the perceived burden was significantly associated with suicidal ideation; and that the interaction between frustrated belonging, perceived burden and suicide capacity was significantly related to a greater number of previous suicide attempts. The experience of DV can frustrate interpersonal needs, thereby increasing the risk of suicidal ideation. DV victims may have a high risk of suicidal ideation due to increased feelings of burden and disconnectedness. First, the perception of a lack of reciprocal caring relationship from one’s partner and social isolation related with the partner’s control, which are probably inherent features of DV, could help explain one’s frustrated belonging. As found in previous studies with a Spanish sample, DV victims repeatedly show greater feelings of loneliness and assess their social network more negatively than non–victimized or occasionally victimized adolescents [75]. Second, suffering experiences of humiliation, detachment or coercion from a partner may increase the perceived burden and self–hatred. Some studies have shown that young women DV victims report emotional distress and a profound self–discontent [76]. In the same vein, another study has found that the public nature of information and distribution of shameful images (difficult to remove but easy to share) in online DV are particularly humiliating experiences for adolescents [17]. Studies such as those conducted by Lamis et al. [36] and Wolford–Clevenger et al. [37] confirm that when the level of frustrated membership is high, the perceived burden correlates with suicidal ideation. Thus, theoretical and empirical reasons exist to expect DV victimization may increase suicidal thoughts and the risk of suicide attempts in victims.

Correlation analyses also found that perceived attachment to parents and peers was positively associated, suggesting a positive link between these two supporting systems. As expected, mediation analyses confirmed the effect of DV on SR, suggesting that DV increases thoughts and suicide attempts. DV also had a direct effect on parental and peers’ attachment, indicating that there are more difficulties establishing quality relationships based on trust, communication, and seeking help. Emotional violence involves humiliation, detachment, isolation and elicits fear and compliance restricting social connections, factors that may contribute to increased SR [77]. This result is in accordance with the association between DV and depressive symptoms, one of the most robust correlates of suicidal ideation. Finally, results show that perceived attachment to parents and peers also decreased SR among adolescents. These results are consistent with studies that find a negative effect of detachment from parents and peers on well–being [63,78].

Indirect effects confirmed the mediation role of parental and peer attachment between DV and SR. Perceived attachment to parents and peers could reduce the effect of DV on SR, suggesting that feeling connected to parents and peers is a powerful buffer against suicidal thoughts since it reduces the emotional negative effects of DV. Findings are also consistent with attachment theory. Parents and peers can be trusted, safe and protective figures [47]. DV victims may perceive parents and peers as sensitive and responsive to their emotional states helping them to reduce their feelings of isolation and anger. As a result, high levels of parental support may protect teens from later developing suicidal symptoms [54]. Additionally, the results of the moderation analyses show that these two attachment figures reduce the effect of dating violence on suicide risk in different ways. High parental attachment reduced more the effect of off–online DV on SR. This type of violence was found to be that which increased suicide risk in a much larger amount. This result suggests that perceptions of secure relationships with parents may be more important than the perception of peer attachment for some measures of mental health [63]. Nevertheless, high perceived peer attachment is that which reduces the effects of offline violence on suicide risk. This result is consistent with authors such as [79] who stress that when young people are faced with a violent relationship they will more frequently seek support among their peers.

The strength of the current study is to explore offline, online and off–online DV and its relationship with suicide within the broader context of family functioning and peer relationships. This study also has relevant practical implications. On the one hand, findings suggest that further studies on DV should cover both online and offline types of DV due to the great impact that suffering both types of DV has not only on suicidal ideation, but on actually having tried to take one’s own life. Results indicated that online and offline DV is common among young couples. The considerable prevalence data from online abuse suggests that the use of ICT may have turned into a new tool for DV toward one’s partner, which previously occurred exclusively in face–to–face interactions. Females who had experienced DV were more likely to report negative feelings in addition to considering and attempting suicide. This study highlights the importance of family and peer systems in suicide prevention. There was less SR when parents and peers supported the victim. Low perceived attachment to parents was associated with greater SR in victims relative to the contribution made by peer attachment. This result suggests that parents play the strongest role in buffering negative feelings and mitigating pain and discomfort associated with DV. Furthermore, it provides evidence that adolescents receive qualitatively different aspects of support from their parents and peers. It could suggest that poor family support may be associated with problems in developing self–reliance in early adolescence. As a result, adolescents may be more vulnerable to suffering DV. Programs that seek to prevent DV should work toward introducing a more secure model of attachment that emphasizes a positive self–concept of oneself and of others and pursuing a more open and fluid communication between parents and adolescents. On the other hand, it is relevant to raise awareness about the role of peers and their influence in DV situations. High parent attachment did not appear to compensate for low peer attachment. This indicates that adolescents need to learn to talk constructively with their peers about DV [80]. This implies that a peer group may provide a supportive and encouraging environment for adolescents in terms of self–expression. Therefore, programs should offer knowledge and tools on how to intervene without increasing the perils for those involved [50]. In sum, communities, parents and other professionals all have a role to play in supporting and informing young people about the risks of dating and guiding them to make healthy and safe choices and decisions.

However, the study has a series of limitations. First, we used self–reported measures for DV, SR and perceived attachment to parents and peers. Thus, social desirability could affect responses regarding sexual violence or suicidal thoughts. Secondly, we used a cross–sectional design, and as a result, it was not possible to infer the exact nature of the relationship between DV and SR. As such, DV may be a consequence rather than an antecedent [43]. Third, selecting the cut–off point as “zero tolerance” may lead to a high percentage of false positives. Fourth, considering that the sample includes an extensive age range (13 to 28), the age variable was controlled in the analyses. However, including this wide range could be a limitation of the study in terms of generalizing results (external validity) to adolescent women who are in an initial and intermediate adolescence phase, and those who are living through adult transition (over 20 years). During this time span, romantic relationships, the role of parents and peers, and suicide risk may vary. This limitation leaves future lines open for analyzing DV and SR and develop specific comparisons according to those age groups. However, evidence strongly suggests that the capacity of young females to detect and label abuse is far from optimal [69] and that being over–cautious in the selection process draws attention to the problem of minimizing abuse. Fourth, in the study we have used the same instrument to measure the relationship with both parents (mother and father). It could be appropriate in future studies to use a measure that differentiates each parent and the role they play as support and attachment figures. Finally, and despite having a significant sample size, it is nevertheless a convenience sample which limits the generalization of results to other contexts. 

## 5. Conclusions

This study attests to the fact that a significant number of female adolescents in this study reported experiencing both off and online DV. While psychological violence is the most common type of face–to–face DV, monitoring and control is the most common type of online DV. As could be expected, DV has negative psychological and emotional effects on victims. Suffering off and online DV can frustrate interpersonal needs and increase the risk of suicidal ideation nearly threefold compared to those who do not report these experiences. This experience has a relevant effect on these young girls increasing the likelihood of attempted suicide by 3.5 times in online DV, by four times for those with offline DV, and by over 10 times in those victims of off-online DV compared to non–victims. The importance of having other people who may comfort you is underlined by the fact that DV victimization and perceived attachment problems with parents and friends are positively related to suicide risk. Perceived functional attachment can act as a buffer for victims against suicidal thoughts and behaviors. Adolescent girls receive qualitatively different aspects of support from their parents and peers. This study confirms the importance of family and peer systems in suicide prevention in DV victims. Future interventions with female adolescents with DV should explore the presence of simultaneous off– and online victimization. Moreover, these female victims of DV could benefit from activities that focus on the perceived positive and safe attachment styles that both parents and peers can provide. For example, by focusing on empowering members of both reference groups to talk constructively about relationships with adolescent girls. Providing a supportive and encouraging environment for self–expression, as well as informing young people about the risks of dating and guiding them to choose healthy options is an important basis for reducing thoughts or behaviors about taking one’s life in victims of DV. 

## Figures and Tables

**Table 1:** Prevalence Dating Violence and Suicide Risk.

Variables	α	Yes		No	
		*n*	*%*	*n*	*%*
Offline Dating Violence ^1^	0.910	899	76.0	284	24.0
Detachment ^2^	0.788	793	65.4	420	34.6
Humiliation ^3^	0.824	436	35.9	777	64.1
Coercion ^4^	0.816	630	51.8	586	48.2
Physical ^5^	0.956	138	11.3	1080	88.7
Sexual ^6^	0.970	372	30.5	846	69.5
Online Dating Violence ^7^	0.924	822	68.8	373	30.4
Monitoring/Control ^8^	0.940	810	66.7	405	33.3
Direct Aggression ^9^	0.826	401	33.3	802	66.7
Offline + Online DV ^10^		697	56.8	458	37.3
Suicide (3 items)	0.774				
Thought about suicide		279	22.7	948	77.3
Told anyone you would take your own life		138	11.2	1089	88.8
Tried to take your own life		98	8.0	1129	92

Notes: Missing data: ^1^ *n* = 44 (3.6%); ^2^ *n* = 14 (1.16%); ^3^ *n* = 14 (1.1%); ^4^ *n* = 11 (.9%); ^5^ *n* = 9 (.7%); ^6^ *n* = 9 (.7%); ^7^ *n* = 32 (2.6%); ^8^ *n* = 12 (1.0%); ^9^ *n* = 24; (2.0%); ^10^ *n* = 72; (5.9%).

**Table 2:** Relationship between Off- and Online DV and Suicidal Ideation and Suicidal Behavior.

Variables	Suicide Thoughts				Attempted Suicide			
	Yes	No			Yes	No		
	*n (%)*	*n (%)*	*X2*	OR (95% CI)	*n (%)*	*n (%)*	*X2*	OR (95% CI)
Offline DV								
Yes	235 (89)	664 (72.3)	31.587 ***	3.11 (2.06, 4.69)	83 (92.2)	816 (74.7)	14.063 ***	4.02 (1.83, 8.81)
No	29 (11)	255 (27.7)			7 (7.8)	277 (25.3)		
Detachment								
Yes	219 (80.5)	574 (61)	35.503 ***	2.64 (1.90, 3.66)	82 (87.2)	711 (63.5)	21.509 ***	3.92 (2.11, 7.27)
No	53 (19.5)	367 (39)			12 (12.8)	408 (36.5)		
Humiliation								
Yes	161 (57.7)	275 (29.4)	74.531 ***	3.37 (2.48, 4.30)	64 (65.3)	372 (33.4)	39.921 ***	3.76 (2.43, 5.80)
No	118 (42.3)	659 (70.6)			34 (34.7)	743 (66.6)		
Coercion								
Yes	180 (64.7)	450 (48)	24.166 ***	1.99 (1.50, 2.62)	65 (67)	565 (50.5)	9.756 **	3.99 (1.28, 3.09)
No	98 (35.3)	488 (52)			32 (33)	554 (49.5)		
Physics								
Yes	60 (21.9)	78 (8.3)	39.299 ***	3.11 (2.15, 4.49)	32 (33.3)	106 (9.4)	50.222 ***	4.79 (2.99, 7.66)
No	214 (78.1)	866 (91.7)			64 (66.7)	1016 (90.6)		
Sexual								
Yes	148 (53.2)	224 (23.8)	87.463 ***	3.63 (2.75, 4.81)	59 (60.2)	313 (27.9)	44.202 ***	3.90 (2.55, 5.96)
No	130 (46.8)	716 (76.2)			39 (39.8)	807 (72.1)		
Online DV								
Yes	217 (81.6)	605 (65.1)	26.079 ***	2.37 (1.69, 3.32)	83 (88.3)	739 (67.1)	18.090 ***	3.69 (1.96, 7.01)
No	49 (18.4)	324 (34.9)			11 (11.7)	362 (32.9)		
Direct Aggression								
Yes	149 (55.6)	252 (27)	76.912 ***	3.39 (2.56, 4.49)	59 (62.8)	342 (30.8)	39.750 ***	3.78 (2.44, 5.85)
No	119 (44.4)	683 (73)			35 (37.2)	767 (69.2)		
**Monitoring/Control**								
Yes	213 (77.5)	597 (63.5)	18.615 *^**^	1.97 (1.44, 2.69)	82 (85.4)	728 (65.1)	16.490 *^**^	3.14 (1.76, 5.61)
No	62 (22.5)	343 (36.5)			14 (14.6)	391 (34.9)		
Off-Online DV								
Yes	193 (92.3)	504 (74.2)	31.072 *^**^	4.19 (2.44, 7.17)	70 (97.2)	627 (76.8)	16.283 *^**^	10.55 (2.56. 44,43)
No	16 (7.7)	175 (25.8)			2 (2.8)	189 (23.2)		

Notes: Suicide thoughts = item N.13; Attempted suicide = item N.15. ** *p* ≤ 0.010; *** *p* ≤ 0.001.

**Table 3:** Partial Correlations between Off- and Online DV, IPPA-P, IPPA-F and Suicide Risk.

Variables	*M*	DS	1	2	3	4	5	6	7	8	9	10	11	12	13	14	15	16	17	18
1. Offline total DV	1.78	2.41																		
2. Detachment	0.69	0.84	0.801 ***																	
3. Humiliation	0.29	0.61	0.834 ***	0.602 ***																
4. Coercion	0.45	0.71	0.818 ***	0.493 ***	0.595 ***															
5. Physics	0.07	0.27	0.526 ***	0.293 ***	0.376 ***	0.433 ***														
6. Sexual	0.27	0.62	0.717 ***	0.365 ***	0.517 ***	0.507 ***	0.351 ***													
7. Online total DV	1.00	1.45	0.678 ***	0.420 ***	0.533 ***	0.722 ***	0.406 ***	0.477 ***												
8. Monitoring/Control	0.81	1.14	0.653 ***	0.414 ***	0.494 ***	0.731 ***	0.367 ***	0.430 **	0.953 ***											
9. Direct Aggression	0.19	0.44	0.595 ***	0.413 ***	0.492 ***	0.529 ***	0.364 ***	0.455 ***	0.768 ***	0.588 ***										
10. Off–Online DV	1.44	0.76	0.462 ***	0.454 ***	0.314 ***	0.400 ***	0.168 ***	0.281 ***	0.376 ***	0.455 ***	0.296 ***									
11. IPPA_P total	4.37	1.72	–0.214 ***	–0.209 ***	–0.156 ***	–0.140 ***	–0.101 ***	–0.168 ***	–0.139 ***	–0.139 ***	–0.149 ***	–0.240 ***								
12. Communication	2.75	0.77	–0.162 ***	–0.164 ***	–0.122 ***	–0.099 ***	–0.047	–0.134 **	–0.077 **	–0.080**	–0.099 ***	–0.194 ***	0.889 ***							
13. Trust	3.36	0.62	–0.223 ***	–0.242 ***	–0.152 ***	–0.140 ***	–0.124 ***	–0.152 ***	–0.145 ***	–0.146 ***	–0.155 ***	–0.255 ***	0.870 ***	0.642 ***						
14. Alienation	1.73	0.59	0.179 ***	0.142 ***	0.137 ***	0.132 ***	0.102 ***	0.157 ***	0.155 ***	0.150 ***	0.143 ***	0.179 ***	–0.846 ***	–0.610 ***	–0.650 ***					
15. IPPA_F total	1.51	0.48	–0.147 ***	–0.096 ***	–0.144 ***	–0.090 **	–0.058 *	–0.160 ***	–0.059 *	–0.054	–0.070 *	–0.109 ***	0.279 ***	0.247 ***	0.218 ***	–0.263 ***				
16. Communication	3.26	0.59	–0.077 **	–0.047	–0.091 ***	–0.040	–0.014	–0.089 **	–0.003	–0.002	–0.025	–0.047	0.186 ***	0.201 ***	0.126 ***	–0.149 ***	0.870 ***			
17. Trust	3.55	0.49	–0.125 ***	–0.069 *	–0.134 ***	–0.085 **	–0.061 *	–0.128 ***	–0.061 *	–0.055	–0.056 ***	–0.099 ***	0.187 ***	0.157 ***	0.167 ***	–0.167 ***	0.869 ***	0.669 ***		
18. Alienation	1.51	0.47	0.177 ***	0.133 ***	0.142 ***	0.109 ***	0.079 **	0.195 ***	0.095 ***	0.089 **	0.102 ***	0.136 ***	–0.339 ***	–0.264 ***	–0.268 ***	0.365 ***	–0.754 ***	–0.441 ***	–0.0505 ***	
19. Suicide Risk	0.41	0.84	0.343 ***	0.293 ***	0.317 ***	0.187 ***	0.167 ***	0.304 ***	0.186 ***	0.171 ***	0.213 ***	0.191 ***	–0.362 ***	–0.278 ***	–0.336 ***	0.344 ***	–0.205 ***	–0.119 ***	–0.128 ***	0.281 ***

* *p* ≤ 0.050; ** *p* ≤ 0.010; *** *p* ≤ 0.001.

**Table 4:** Moderation Analyses: Conditional effects of Offline on Suicide Risk at Different Values of the IPPA–P and IPPA–F.

Predictors	Suicide Risk					
					95% CI	
	***b***	***SE***	***t***	***p***	***LL***	***UL***
Offline DV	1.024	0.274	3.738	0.001	0.486	1.561
IPPA–P	–0.139	0.018	–7.761	0.0001	–0.174	–0.104
Offline DV × IPPA–P	–0.047	0.035	–1.341	0.180	–0.116	0.022
IPPA–F	–0.037	0.023	–1.638	0.102	–0.082	0.007
Offline DV × IPPA–F	–0.114	0.045	–2.508	0.012	–0.203	–0.025
Age	–0.008	0.008	–0.990	0.322	–0.024	0.008
**Conditional effects of Offline DV at different values of the moderators:**						
**IPPA–F**	**IPPA–P**	***Effect***	***SE***	***p***	**95% CI**	
Low (4.00)	Low (2.75)	0.438	0.104	0.0001	0.235	0.642
	Medium (4.75)	0.344	0.087	0.0001	0.174	0.515
	High (6.00)	0.285	0.103	0.005	0.084	0.487
Medium (5.58)	Low (2.75)	0.258	0.092	0.005	0.077	0.439
	Medium (4.75)	0.164	0.054	0.002	0.058	0.270
	High (6.00)	0.105	0.067	0.115	–0.026	0.236
High (6.50)	Low (2.75)	0.154	0.109	0.160	–0.061	0.368
	Medium (4.75)	0.059	0.071	0.400	–0.079	0.198
	High (6.00)	0.001	0.075	0.994	–0.147	0.148

*R^2^* = 0.165, *F*_(6,1176)_ = 38.684, *p* = 0.0001; Notes: Offline DV *N* = 1183; IV: Offline DV; Moderators: IPPA–P (parents) and IPPA–F (friends); DV: Suicide Risk.

**Table 5:** Moderation Analyses: Conditional effects of Online on Suicide Risk at Different Values of the IPPA–P, IPPA–F.

Predictors	Suicide Risk					
	*b*	*SE*	*t*	*p*	95% CI	
					*LL*	*UL*
Online DV	0.581	0.228	2.544	0.011	0.133	1.030
IPPA–P	–0.144	0.017	–8.570	0.0001	–0.177	–0.111
Online DV × IPPA–P	–0.043	0.033	–1.293	0.196	–0.107	0.022
IPPA–F	–0.068	0.020	–3.343	0.001	–0.108	–0.028
Online DV × IPPA–F	–0.039	0.041	–0.963	0.335	–0.119	0.041
Age	–0.009	0.008	–1.123	0.262	–0.025	0.007

*R^2^* = 0.162, *F*_(6,1188)_ = 38.215, *p* = 0.0001; Notes: Online DV *N* = 1195; IV: Online DV; Moderators: IPPA–P (parents) and IP+PA–F (friends); DV: Suicide Risk.

**Table 6:** Moderation Analyses: Conditional effects of Off-Online on Suicide Risk at Different Values of the IPPA–P, IPPA–F.

Predictors	Suicide Risk					
	*B*	*SE*	*t*	*p*	95% CI	
					*LL*	*UL*
Off–Online *DV*	1.250	0.327	3.818	0.0001	0.607	1.892
IPPA–P	–0.108	0.023	–4.797	0.0001	–0.153	–0.064
Off–Online DV × IPPA–P	–0.096	0.044	–2.188	0.029	–0.183	–0.010
IPPA–F	–0.046	0.027	–1.677	0.094	–0.099	0.008
Off–Online DV × IPPA–F	–0.095	0.005	–1.738	0.082	–0.201	0.012
Age	–0.016	0.009	–1.711	0.087	–0.035	0.002
**Conditional effects of the Off–online DV at different values of the moderators:**						
**IPPA–P**	**IPPA–F**	***Effect***	***SE***	***p***	**95% CI**	
					***LL***	***UL***
Low (2.50)	Low (4.00)	0.630	0.135	0.0001	0.365	0.895
	Medium (5.50)	0.488	0.128	0.0001	0.237	0.740
	High (6.50)	0.394	0.150	0.009	0.099	0.689
Medium (4.75)	Low (4.00)	0.413	0.103	0.0001	0.211	0.615
	Medium (5.50)	0.271	0.067	0.0001	0.139	0.403
	High (6.50)	0.177	0.089	0.046	0.003	0.351
High (6.00)	Low (4.00)	0.293	0.122	0.016	0.054	0.532
	Medium (5.50)	0.151	0.080	0.060	–0.006	0.308
	High (6.50)	0.056	0.090	0.533	–0.121	0.234

*R^2^* = 0.172, *F*_(6,881)_ = 30.466, *p* = 0.0001; Notes: Off–online DV *N* = 888; IV: Off–online DV; Moderators: IPPA–P (parents) and IPPA–F (friends); DV: Suicide Risk.
